# Supplementary material for: Laccase-Mediated Oxidation of Phenolic Compounds from Wine Lees Extract towards the Synthesis of Polymers with Potential Applications in Food Packaging
Source: Biomolecules. 2024 Mar 8;14(3):323. doi: 10.3390/biom14030323 (PMC10968617; doi:10.3390/biom14030323)
Supplement: Supplementary file 1 [file biomolecules-14-00323-s001.zip › biomolecules-2880015-supplementary.pdf]

biomolecules

*Supplementary Materials*

# Laccase-mediated oxidation of phenolic compounds from wine lees extract towards the synthesis of polymers with potential applications in food packaging

**Panagiotis E. Athanasiou [1], Christina I. Gkountela [2], Michaela Patila [1], Renia Fotiadou [1], Alexandra V. Chatzikonstantinou[1], Stamatina N. Vouyiouka [2] and Haralambos Stamatis [1,*]**

[1] Laboratory of Biotechnology, Department of Biological Applications and Technology, University of Ioannina, 45110 Ioannina, Greece
[2] Laboratory of Polymer Technology, School of Chemical Engineering, National Technical University of Athens, Zographou Campus, 15772 Athens, Greece
* Correspondence: hstamati@uoi.gr (H.S.)

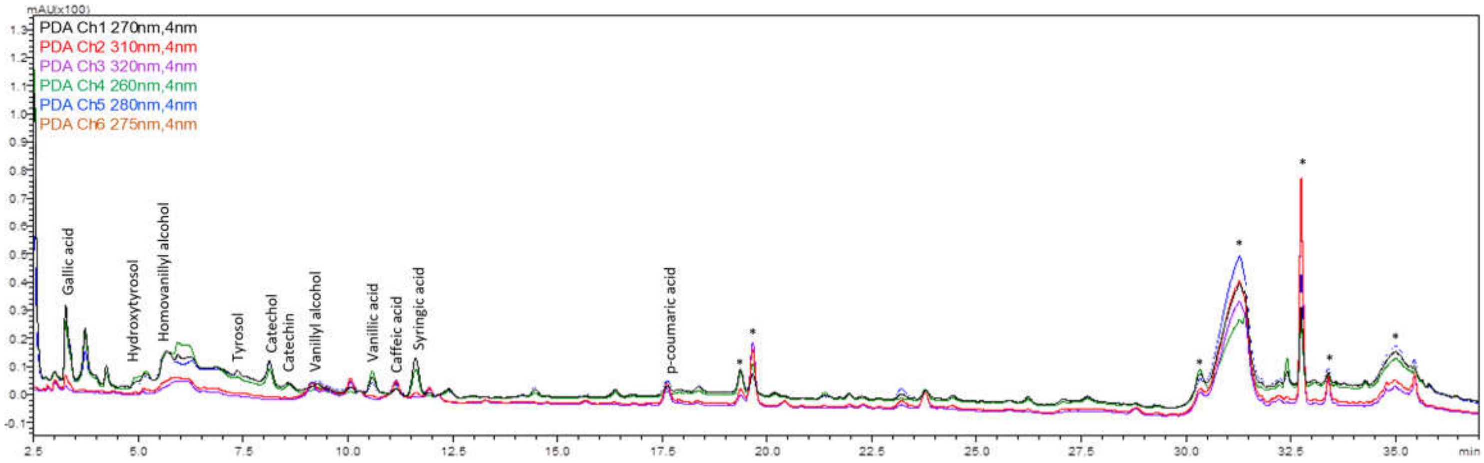


**Figure S1**. HPLC chromatogram of the reaction's supernatant before the polymerization process. Individual peaks of known phenolic compounds are marked by their respective names. Asterisk indicates individual unknown compounds with characteristic aromatic-like spectra.

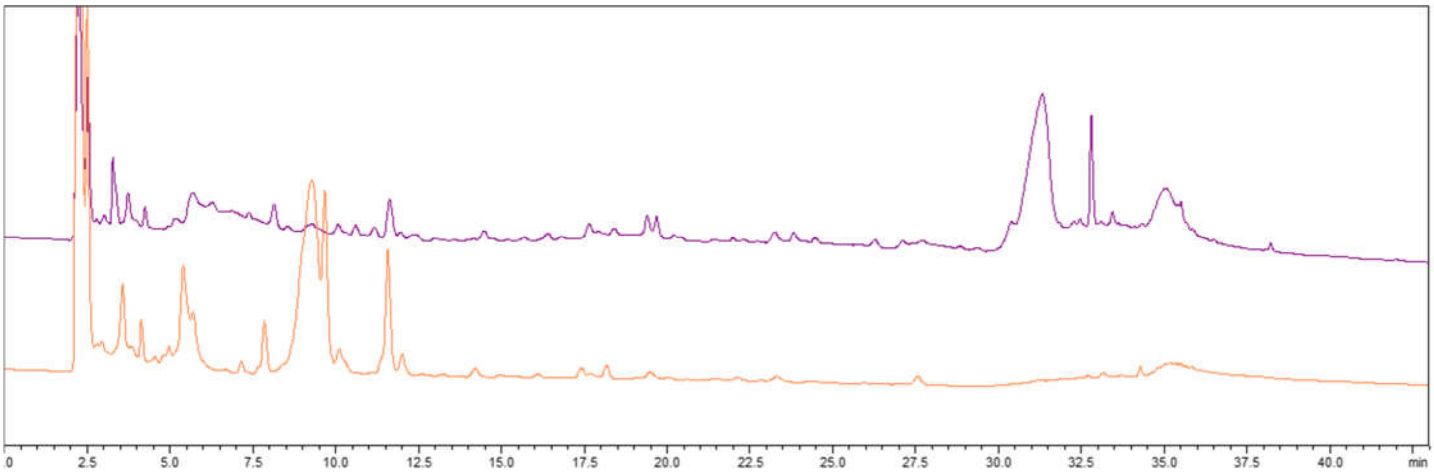


**Figure S2**. HPLC chromatograms at 280 nm of the WLE before and after TvL treatment.

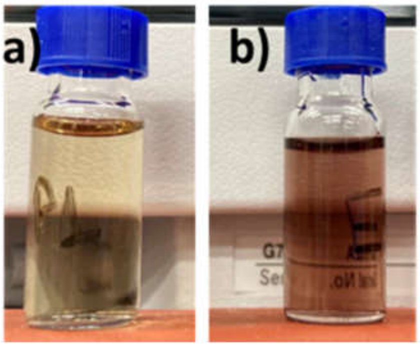


**Figure S3**. (a) The polymeric product P1h and (b) the extract dissolved in DMF.

**Table S1**. Thermal properties defined via TGA of the extract and the P1h and P24 h synthesized polymers.

| Sample | $T_{d,5\%}$ (°C) | $T_{d1}$ (°C) | $T_{d2}$ (°C) | Residue (%) |
|---|---|---|---|---|
| Extract | 164 | 194 | - | 35 |
| P1h | 186 | 238 | 308 | 29 |
| P24h | 192.5 | 218 | 271 | 34 |

**Table S2**. Thermal properties defined via DSC of the extract and the P1h and P24 h synthesized polymers.

| Sample | Endotherm 1 (°C) | $\Delta H_1$ (J·g$^{-1}$) | Endotherm 2 (°C) | $\Delta H_2$ (J·g$^{-1}$) | Endotherm 3 (°C) | $\Delta H_3$ (J·g$^{-1}$) | Endotherm 4 (°C) | $\Delta H_4$ (J·g$^{-1}$) |
|---|---|---|---|---|---|---|---|---|
| Extract | 31 | 1 | 137 | 26 | 201 | 27 | 283 | 32 |
| P1h | 38 | 6 | 122 | 9 | 272 | 9 | 356 | 5 |
| P24h | 40 | 5 | 123 | 10 | 271 | 23 | 344 | 3 |

**Table S3**. Antioxidant activity (%) of the formed polymeric products (tested concentration: 100 μg mL$^{-1}$)

| | Antioxidant activity (%) | |
|---|---|---|
| Sample | ABTS | DPPH |
| P1h | 82.8 ± 1.9 | 93.1 ± 0.4 |
| P2h | 74.7 ± 2.8 | 87.4 ± 1.0 |
| P6h | 73.8 ± 2.5 | 84.8 ± 0.5 |
| P24h | 70.6 ± 1.5 | 84.9 ± 0.2 |
